# Real Time QRS Detection Based on M-ary Likelihood Ratio Test on the DFT Coefficients

**DOI:** 10.1371/journal.pone.0110629

**Published:** 2014-10-30

**Authors:** Juan Manuel Górriz, Javier Ramírez, Alberto Olivares, Pablo Padilla, Carlos G. Puntonet, Manuel Cantón, Pablo Laguna

**Affiliations:** 1 Department of Signal Theory, Telematics and Communications, CITIC, University of Granada, Granada, Spain; 2 Department Computer Architecture and Computer Technology, CITIC, University of Granada, Granada, Spain; 3 Department of Informatics, University of Almería, Almería, Spain; 4 Department of Electronic Engineering and Communications, University of Zaragoza, Zaragoza, Spain; University of Rome Tor Vergata, Italy

## Abstract

This paper shows an adaptive statistical test for QRS detection of electrocardiography (ECG) signals. The method is based on a M-ary generalized likelihood ratio test (LRT) defined over a multiple observation window in the *Fourier domain*. The motivations for proposing another detection algorithm based on maximum *a posteriori* (MAP) estimation are found in the high complexity of the signal model proposed in previous approaches which i) makes them computationally unfeasible or not intended for real time applications such as intensive care monitoring and (ii) in which the parameter selection conditions the overall performance. In this sense, we propose an alternative model based on the independent Gaussian properties of the Discrete Fourier Transform (DFT) coefficients, which allows to define a simplified MAP probability function. In addition, the proposed approach defines an adaptive MAP statistical test in which a global hypothesis is defined on particular hypotheses of the multiple observation window. In this sense, the observation interval is modeled as a discontinuous transmission discrete-time stochastic process avoiding the inclusion of parameters that constraint the morphology of the QRS complexes.

## Introduction

One of the most relevant waveforms in the electrocardiogram (ECG) is the QRS complex since it has been used in several medical applications [1] such as noise cancelation [2], the automated determination of the heart rate [3] or computer-based arrhythmia monitoring [4]. The QRS ECG segment reflects the electrical activity during ventricular contraction, thus the time of its occurrence as well as its shape provide relevant diagnostic and prognostic information in clinical practice [5]–[7].

In the past decades several approaches to QRS detection based on different paradigms have been successfully proposed. Examples of such approaches are based on the field of artificial neural networks [8], genetic algorithms [9], wavelet transform [10] or filter banks [11], analyses of signal parameters such as slope, amplitude and width [12], as well as other heuristic [13] and non linear transforms. Most QRS detectors have been developed following a three-step structure [3], that is, a linear filter suppressing noise and artifacts followed by a nonlinear transformation for signal enhancement. The output of these two stages is then fed to a third decision rule stage for detection. The main target of this paper is focused on the third stage, therefore the proposed method could be used in combination with detectors described in the literature which have been developed from *ad hoc* reasoning and experimental insight.

Up to our knowledge the first approach based on maximum *a posteriori* (MAP) estimation for QRS detection was proposed in [14]. In the latter work a complex mathematical model in the time domain was introduced to find several ECG parameters such as amplitudes, widths or arrival times, which provide an appropriate fit to that model using a pre-defined *matched filter*. As the authors acknowledge, this method was computationally unfeasible [14], thus additional simplifications and approximations on the MAP estimation were needed to be introduced to reduce the computation time [15], but still the method could not be considered as a real time approach, i.e. the estimation of arrival times are not necessarily found in temporal order [15]. This problem can, of course, be solved if the size of the observation window is reduced, as shown in the experimental part of this paper where this method is analyzed as a baseline. This is mainly motivated by the *long-term* observation window of the model which assumes that the observation vector contains an unknown number *q* of pulse-shaped waveforms.

On the other hand, the asymptotic properties of the Discrete Fourier Transform (DFT) coefficients [16] could be as well analyzed in the definition of the signal model, that is they are defined as Gaussian variables. If these assumptions are considered in context, an effective and *real-time* M-ary Likelihood Ratio Tests (LRT) detector could be derived with a lower number of parameters to be estimated, i.e. only the variances of the noise and desired signal. In this sense, we showed recently [17] that incorporating contextual information from preceding and succeeding samples, and multiple hypotheses in the LRT, reports benefits for signal detection in other fields of research such as speech processing or voice activity detection. This paper analyzes this signal model together with other innovations, showing a novel QRS detector that extends the number of hypotheses of the M-ary LRT in a multiple observation window.

The rest of the manuscript is organized as follows. Section shows a general description of the signal model and the detector structure. Topics such as the definition of the M-ary LRT, the partial and global hypotheses that are considered in the test, and a revised maximum *a posteriori* (MAP) statistical test are presented and discussed. Several examples are also discussed as well as the influence of the model parameters in the detector performance. Moreover, in this section a robust method for statistical parameter estimation, i.e. the PSDs of the QRS and noise processes, is shown based on the minimum mean-square error (MMSE) estimator [16]. Section 0.0.1 analyzes the proposed detector together with an approximate statistical LRT related to previous approaches in speech recognition such as [17]–[19]. Section 0.0.1 is devoted to the experimental framework including the discrimination analysis and the QRS detection performance evaluation. All the experiments are carried out on the MIT-BIH Arrhythmia standard Database [20]. Finally, section 0.0.1 summarizes the conclusions of this work.

## Adaptive QRS detection based on MAP LRT

### Signal Model

The ECG signal is modeled as a discrete-time stochastic process [15]; typically, the observation signal for a real time QRS detector, including at most 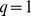 pulse-shaped waveforms, is given by:
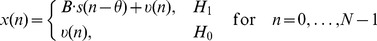
(1)


where 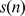 is the QRS complex with known morphology (pulse-shaped waveform), arrival time 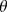, amplitude 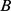 and width 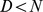 which is corrupted by a stationary, white, Gaussian process 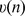 with variance 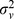. Furthermore, 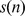 is considered to be composed of two identical waveforms 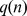, one of which is shifted 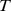 samples in time and has opposite sign:
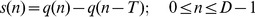
(2)


In addition, the temporal parameters of this model are considered as discrete/continuous random variables with known probability densities which are relevant for subsequent ECG analysis [14], [15]. Based on the observed signal 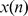 the structure of the MAP estimator is derived by maximizing the log-likelihood function in [15], which depends on the previously defined parameters and relies on the Gaussianity of the noise.

Some of the drawbacks associated with the present model are: the large amount of parameters to be tuned during the design and test of the system; the noise is neither stationary nor ergodic, i.e. the occurrence of noise is due to different waveforms such as P or T waves, from myoelectric origin or transient artifacts; some changes in the QRS morphology could arise from physiological origin or technical problems being unlikely to be effectively modeled by just an amplitude value; etc. Therefore, obtaining the detector structure by maximizing the log-likelihood function as the one in [15], requires the estimation of parameters that are essentially time dependent since probability distributions could be time-varying.

Due to the above-mentioned reasons another simpler statistical model is used in this paper which is based on theoretical findings in the Fourier analysis [21]. The Fourier expansion coefficients of the observed signal are assumed to be statistically independent Gaussian random variables:
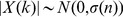
(3)


These coefficients are obtained by decomposing the signal into overlapped frames each of size 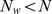 with a 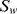-sample window shift, where *N* is total number of samples of the signal, and by computing the *J*-point windowed DFT spectral representation on a frame by frame basis:
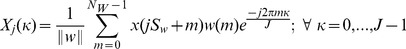
(4)


where 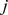 denotes the frame index, 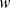 represents the window (typically a Hamming window to reduce the correlation between widely separated spectral components) and 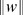 is its norm. Thus, 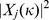 is a consistent estimation of the power spectral density (PSD) of the signal.

In the Fourier domain the observation window can be rewritten as:
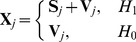
(5)


Thus, once the window size 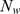 is selected, the channel can be described as a vector sequence 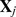 that alternates between two possible states, i.e. presence or absence of pulse-shaped waveforms (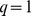 and 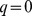 respectively). Assuming that the total number of observations (i.e. frames) is 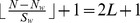 and the number of signal observations is 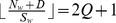, the partial observation vectors can be reindexed and grouped into a global observation matrix (from now on *buffer*):
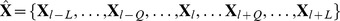
(6)


The content of the buffer, assumed symmetric without loss of generality, is shifted one position to the left in each step of the algorithm so the new feature vector obtained after the analysis of the current analysis window is inserted in the (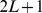)-th position. Based on this signal model the detector now can formulate a binary decision about the presence or absence of the QRS complex in the central frame stored in the central position (frame 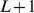) without loss of generality, using the 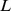 preceding observations 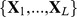 and the 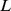 succeeding observations 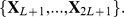 Consequently, any algorithm using this observation model exhibits only an 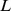-frame computational delay so that the decision over the (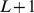)-th frame of the signal is only available after the (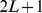)-th frame has been analyzed.

### Detector Structure based on MAP M-ary LRT

Given the signal model in equations 3 to 6, the probability for each observation vector can be evaluated under binary hypothesis testing as:
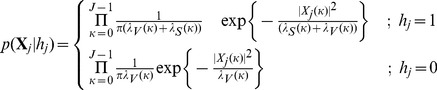
(7)


where 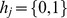 is the partial hypothesis, that is, the presence or absence of the QRS complex in the observation vector 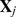; and 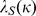 and 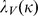 are the PSDs of the QRS and noise processes, respectively, which are estimated using the Ephraim and Malah minimum mean-square error (MMSE) estimator [16]. The observation vectors 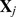 in the buffer 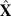 are assumed to be statistically independent, thus the conditional probability conditioned on the global hypothesis 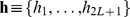, can be calculated by
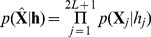
(8)


The independence approximation between observation frames that is followed by many authors [15], [22] is considered and analyzed in a previous work [22]. Nevertheless the correction introduced in the latter paper requires a very complex model for the observation probabilities in the simplest case (jointly Gaussian observation frames), and does not substantially alter the performance of the detector, although the overlap between observation frames introduces a significant correlation between them.

Let 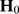 and 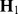 be the set (or the matrices) of hypotheses (or *states*) 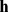 which define the absence or presence of the QRS complex in the buffer, respectively, and follow the selection criteria:
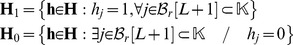
(9)


where 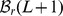 stands for the closed ball of radius *r* centered at 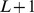 in the space of integers 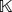. Thus, the presence or absence of the QRS complex depends on the partial hypotheses formulated on the central frames of the buffer. Taking into account this definition, the joint probabilities 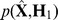 and 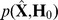 can be obtained by:
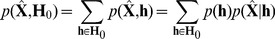
(10)

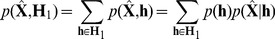
(11)


where 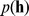 is the *a-priori* probability of hypothesis 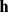 (see Appendix S1 for the calculation of these probabilities). As readily shown from (10) the maximization of the likelihood function avoids the estimation of the pdf for several parameters related to the desired signal features (amplitude, width, etc.) [14] or the parameter selection to define a subset of matched filters that better maximize this function [15]. However, it requires to estimate the *a priori* probability of the states, that can be easily measured analyzing an ECG template (see section 0.0.1 and Appendix S1).

Finally, in order to detect the QRS complex, the MAP optimum criterion is defined to be an M-ary LRT (with 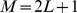) as follows:
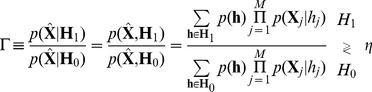
(12)


where the decision threshold 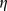 is used to tune the operating point of the detector. Thus, the largest conditional probability is selected by computing the weighted probability of the states defined in (9). If (12) is approximated by taking the maximum log value of the hypotheses, a revised statistical test can be defined in matrix form removing the summation symbols as:
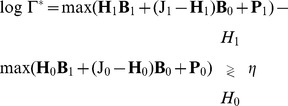
(13)


where 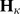 is the 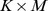 row-wise matrix of states 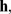 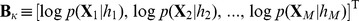, 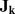 is the 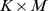 matrix of ones and 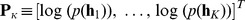, 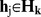 is the column vector of the logarithmic *a priori* probabilities of the hypotheses in 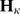. The value 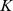 depends on how the selection criteria in (9) are defined.

#### Examples

For 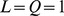 and 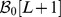, i.e. a ball with radius 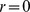, for the selection criteria (only the central frame defines the hypothesis), the 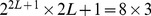 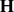 matrix is defined as:
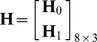



where 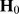 and 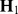 are given by:
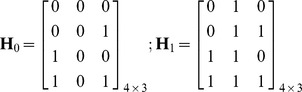



In this case only the central hypothesis defines the presence or the absence of the QRS complex. The selection criteria could be modified in order to be more conservative in the detection of the QRS complex by selecting 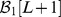 (a ball with radius 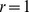), then:
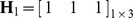



and 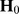 is a 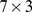 matrix defined by the rest of states which lie out of the ball.

### Estimation of Statistical Parameters

In a real time QRS detector the processing to be performed on the incoming signal 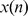 is divided into two phases: i) delimitation of the observation window that should *not exceed the size of the ECG signal period*, i.e. it should not include two QRS complexes; ii) estimation of the model parameters in (7), i.e. 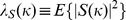 and 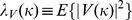. Unlike other approaches we use the same detector structure with the same parameter values for both estimations.

#### Noise Spectrum Estimation

An initial model for the noise spectrum should be determined from the incoming signal. To this purpose fiducial points are computed for a few initial periods of the ECG signal following the procedure described in [23]. Once the isoelectric line is determined the noise spectrum 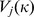 is backward computed from these knots and smoothed by averaging [16]. Moreover the noise spectrum is then updated, in a similar fashion of the recursive averaging method proposed in [16], during the *non-QRS* periods (determined by the detector) by means of a 1st order IIR filter on the smoothed spectrum:
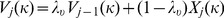
(14)


where 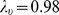.

#### QRS Spectrum Estimation

The clean QRS spectrum is estimated by combining smoothing, spectral subtraction and conventional two-stage mel-warped Wiener filter design [24]. The latter attempts to remove additive noise throughout two filtering stages: the first stage coarsely reduces noise and whitens residual noise; the second stage removes any residual noise.
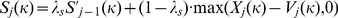
(15)


where 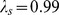. Then the Wiener filter 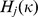 is designed as:
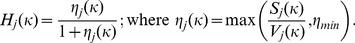
(16)


where 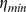 is selected so that the filter yields a minimum attenuation of 20 dB. Finally the clean QRS spectrum is computed as:
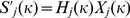
(17)


This Wiener filter design process is repeated twice [24]. With these operations we derive the ML estimators of the 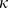-th signal spectral component variance (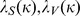) in the *j*-th analysis frame which have been successfully used in other fields such as speech enhancement [16].

The use of the adaptation presented in equation (15) similar to the one in equation (14) allows that the spectrum models can not be affected during failure detection segments, i.e. 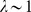.

This kind of adaptations have been successfully applied in other fields such as voice activity detection in speech recognition [20] where the samples rates and spectral width of the signal of interest are higher than the ones in ECG signal processing.

## Analysis of the proposed QRS detector

In this section several aspects of the proposed algorithm are described and analyzed. In particular, we derive an approximation of the M-ary LRT of equation 13 to validate its usability to discriminate QRS frames from noise frames. Moreover, the application on several examples of the analyzed database [20] and the estimation of the *a-priori* probabilities are presented for further analysis.

### *A-priori* probabilities of the states

**Figure 1:**
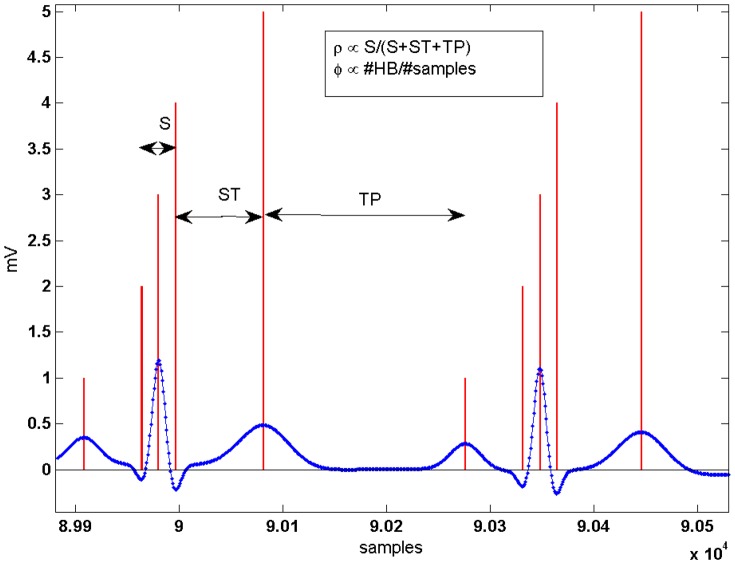
ECG synthetic signal generated for the calculation of *a priori* probabilities [26]. Note HB: heartbeats.

The *a-priori* probabilities of the states 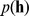 that are necessary to evaluate equation 13, can be calculated in terms of the probability of QRS segments (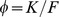, where 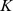 is the total number of QRS blocks, i.e. heartbeats in the signal and  the total number of observation frames) and the *a priori* probability of QRS frames (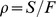 where 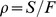 is the total number of QRS frames) as shown in the appendix. These probabilities can be experimentally assessed by the use of manually segmented ECG databases where the proportions for the different ECG segments are available. In this paper an ECG template is used to estimate these probabilities based on the standard test waveforms specified in ANSI/AAMI EC13:1992 [25]. Following this recommendation a synthesized ECG signal was generated [26] with the following parameters:

- ECG sampling frequency: 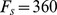 Hz.
- Heart rate mean: 60 bpm.
- LF/HF ratio: 0.5.
- Number of heartbeats: 256 with standard deviation 1 bpm.

obtaining the values 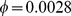 and 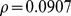 (see figure 1). These probabilities could, of course, be better adjusted using a real manually-segmented ECG record, however the model is performing well enough with these approximate values as it is later shown in the experimental part.

### Approximate log M-ary LRT estimation

**Figure 2:**
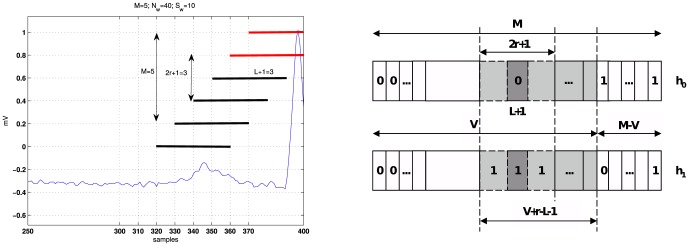
Hypothesis considered for the derivation of the approximate M-ary LRT and its expected value. Left: Example of ECG segment (blue line) and its observation window composed of QRS (red line) and noise (black line) frames (*M* = 5 and *r* = 1). Right: The most probable hypotheses in 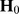 and 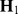 for a transition as shown in left figure.

For a simplification of (13) a particular transition is analyzed (see figure 2). This corresponds to a situation in which 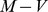 (being V the number of noise samples) observations in the buffer of size 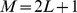 are QRS frames from a total of 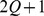 QRS frames. The most probable hypotheses in 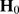 and 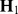, denoted by 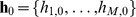 and 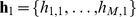 respectively, are evaluated by taking the max logarithms in (12):



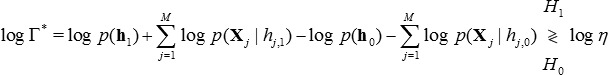



or equivalently for 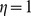:
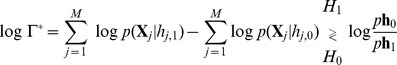
(18)


Removing the partial states of 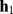 and 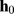 in common that is 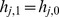 it leads to:
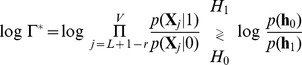
(19)


By defining the subset 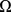 of indexes where 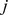 is evaluated (subframe 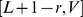 which appears shaded in light gray in figure 2(b)) and substituting equation (7) in the previous equation, the decision rule is finally defined as
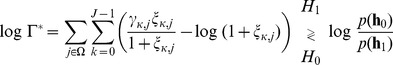
(20)


where 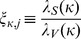 is the *a-priori* SNR for the 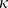-th band and  denotes the *a-posteriori* SNR for the 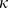-th band at the *j*-th frame of the buffer [27]. In addition, a scaled (normalized in length) decision rule independent of 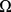 and 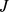:
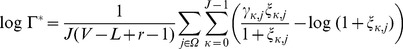
(21)


is preferred, where 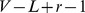 is the cardinality of 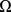. This statistical test can be understood as an average of the decision criterion over the selected frames present in the buffer. Finally, from (24) the expected value can be computed:
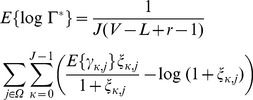
(22)


By using
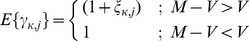
(23)


and assuming stationary white noise and signal models (the SNRs are constant for all the frequency bands 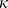) it yields:
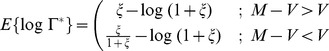
(24)


**Figure 3:**
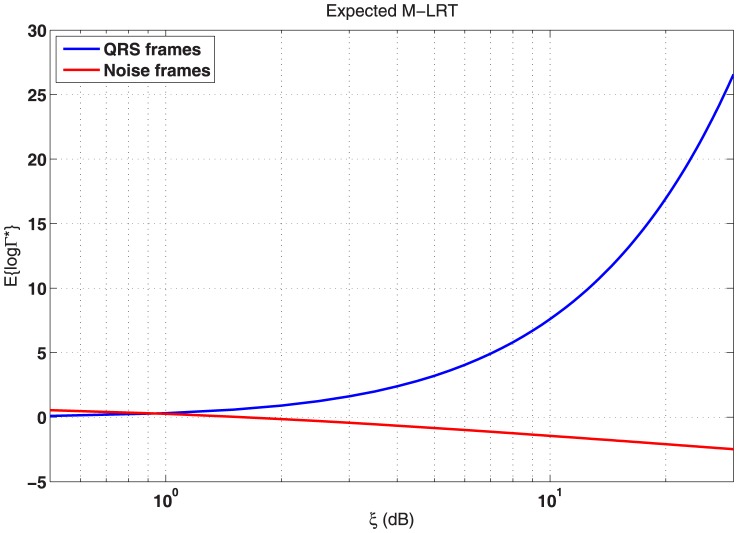
Expected value for the M-ary LRT vs the *a priori* SNR in the detection of a step change.

As shown in Figure 3 under this naive approximation based on a Gaussian process the proposed M-LRT may effectively discriminate between QRS and noise frames for a wide range of SNRs during step transitions in the observation window.

### Additional improvements

A significant improvement will now be discussed by the use of additional parameters in the maximization of the probability log-function in a similar fashion of the models proposed in previous MAP detectors. In particular, we consider 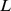 as an independent discrete random variable with uniform pdf 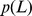 in the interval 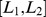, where 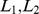 are positive integers. With this innovation the maximization in (13) can be rewritten as:
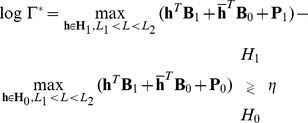
(25)


where 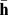 is the logical complement of 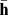. The benefit of the present modification consists in adjusting the window size of the observation interval to the most probable hypotheses. The calculation of this modification is not time consuming for model orders 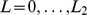, where 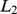 is the target model order, since they are required for the computation of the overall log function and can be evaluated recursively [19]. Let 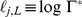 be the 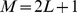-order log-probability function at *j*th frame as shown in Eq. 13, then the recursion in the model order can be evaluated as:
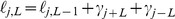
(26)


where 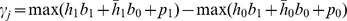, 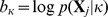 and 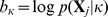; the recursion in time can be evaluated as:
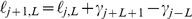
(27)


### Evaluation on real ECG segments

**Figure 4:**
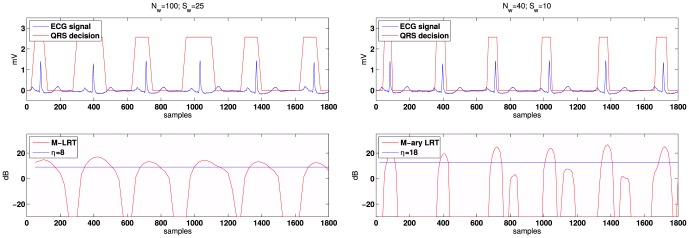
Patient 101 of the MIT-BIH arrhythmia database [20] sampled with

Figure 4 shows two examples of the database [20] and the results of the proposed QRS detector with the same proportion between the length of the window and the length of the overlap ()25%). The selection of the size of the window influences the performance of any detector since it controls the amount of information processed in the test. Typically the QRS complex lasts for about  ms, thus a suitable selection for the observation window is 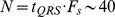 samples.

**Figure 5:**
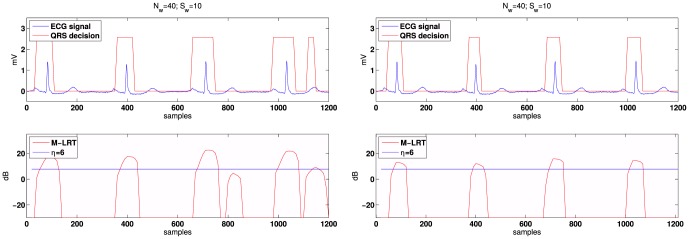
Patient 101 of the MIT-BIH arrhythmia database [20] sampled with 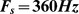. In both cases a 25% overlap between observation windows is selected. The M-ary LRT test is performed with M = 5, order *L* = 2. Left: *r* = 0. Right: *r* = 1 Note how the false alarm in in the last T segment is removed.

On the other hand, the selection criteria are analyzed in figure 5. The decision function of the detector is plotted for *r* = 0 and *r* = 1 radii, where the benefits of the restrictive conditions imposed in (9) for *r* = 1 are highlighted. Under these conditions the detector removes possible false alarms that occur in peaked T segments. Using *r* = 1 and an overlap of 30 samples is a suitable choice taking into account the duration of the QRS segment (∼40 samples). Therefore, this configuration will be used in the experimental part.

## Experiments and Results Discussion

The proposed detector was mainly evaluated in terms of the ability to discriminate between QRS and non-QRS periods at different noise scenarios and SNR levels. All the methods including the matched filter MAP detector as well as the proposed M-ary LRT were evaluated under the same conditions, that is, the same amount of information is available for the segment assessment. In our method the noise reduction method based on the Ephraim and Malah estimator [16] was used for estimating the *a priori* SNR, and an adaptive threshold update enables the effective tuning of the operating point for the wide range of SNR conditions.

### The MIT-BIH database

Several standard ECG databases are available for the evaluation of software QRS detection algorithms [3]. The application of our MAP method on any of these well-annotated and validated databases provides reproducible and comparable results. In addition, these databases satisfy the above-mentioned conditions under which the method should be tested on, that is, they should contain a large number of selected signals representative for the large variety of ECGs, SNRs, as well as signals that are rarely observed but clinically important.

One of these databases is the MIT-BIH database [20], provided by MIT and Boston's Beth Israel Hospital, which consists of ten databases for various test purposes; i.e., the Arrhythmia Database, the Noise Stress Test Database, the Ventricular Tachyarrhythmia Database from Creighton University Cardiac Center, the ST Change Database, the Malignant Ventricular Arrhythmia Database, etc. The first three MIT-BIH databases are required by the ANSI for testing ambulatory ECG devices.

**Figure 6:**
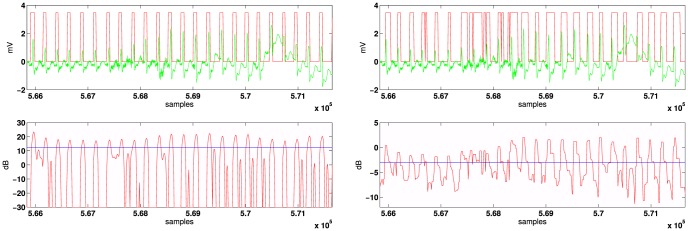
ECG signal in green line (record 108 containing several abnormal shapes, noise and artifacts). Left: MAP decision in red line based on M( = 3)-ary LRT. Right: A real time implementation of the matched filter-based method [15].

The experiments in this paper focus on the Arrhythmia Database which contains 48 half-hour excerpts of two-channel ambulatory ECG recordings, obtained from 47 subjects studied between 1975 and 1979. The recordings were digitized at 360 samples per second per channel with 11-bit resolution over a 10 mV range where several cardiologists independently annotated each record [20], altogether there are about 116137 QRS complexes. While some records contain clear R-peaks and few artifacts (e.g., records 100-107), for some records the detection of QRS complexes is very difficult due to abnormal shapes, noise and artifacts (e.g., records 108 and 207) as shown in figure 6. Note the different decision range for both detectors and the benefits of the proposed QRS decision (Fourier domain versus time domain).

### Performance Measures

The usage of software QRS detection algorithms in medical devices requires the evaluation of the detection performance on standard databases. According to [28], essentially two parameters should be used to evaluate the algorithms; that is,
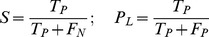
(28)


where 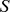 denotes the sensitivity, 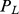 the positive likelihood, 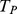 the number of true positive detections, 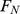 the number of false negatives, and 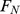 the number of false positives.

Using these measures two MAP decision methods are evaluated on the standard MIT-BIH database in order to get comparable and reproducible results. The former is based on a real time implementation of the matched-filter proposed in [14]. This approach is applied to an observation window comparable with the one used by the other QRS MAP detector (delimitation of the observation interval), thus the method is converted into a real time detector provided that the application of the original approach to the MIT-BIH records is computationally unfeasible. The latter approach is the M-ary LRT-based detector proposed in this paper. Furthermore, we are focussing our attention in the decision rule stage, that is, the methods based on MAP decision, since they mean a general framework and could be used in conjunction with other approaches such as linear filtering, non-linear transformations or heuristics based procedures for the same detection problem.

The model parameters are selected with values 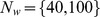 and 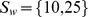 samples, respectively. The order of the model is selected to be 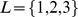, therefore the size of the observation interval is 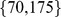 up to 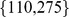 samples. Note that the typical P-QRS-T interval duration is about 530 ms (∼190 samples at 360 Hz) thus the last value is clearly out of this bound. The matched filter used as the baseline method is defined in the time domain as a *perfect replica* of a QRS template using a synthesized ECG signal. Thus, the classical method has been design under the more favorable conditions by using a time-reversed template of the waveform. The maximization of the log function is carried out on the arrival time 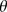 among all possible values in the observation window.

The results of this comparison are shown in table 1, where the 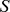 and the 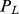 of the proposed and the baseline methods at the operation point are shown. This table summarizes the average hit-rates for all the noises and SNR conditions present in the database of the previously analyzed methods. It is clearly shown that, while the revised method yields similar QRS detection accuracy when compared to the matched filter based detector [14] at low model order, it exhibits an improved accuracy in detecting QRS periods when the order is increased, i.e *M* = 7. The improvements are especially important for poor SNRs and the presence of artifacts or abnormal QRS shapes as shown in figure 6. By using the other combination in the delimitation of the observation interval, i.e. 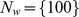 and 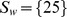 we obtain similar results to those explained before, that is, increasing the model order provides an increase in the detection performance except when *M* = 9 since the observation interval does not fulfill the assumptions held in section 0.0.1, i.e. it contains 325 samples (∼900 ms) thus two QRS complexes may be included in it. As a conclusion it is shown that the M-ary LRT method yields a significant improvement in *S*, and in 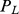 when the model order is greater than three *M*>3 and provides similar results as the trade-off between those measures when compared to the baseline. Moreover, from this analysis the proposed detector scheme for *M* = 5 achieves the best compromise among the different detectors tested. It yields good results in detecting QRS and non-QRS periods and exhibits a very slow performance degradation at unfavorable noise conditions in QRS detection.

**Table 1:** Operation points for the MAP based QRS detectors. Average and deviation of 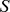 and 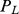 (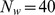, 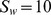).

	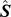	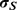	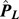	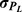
**1-ary LRT**	**0.9386**	**0.0852**	**0.9105**	**0.1201**
**3-ary LRT**	**0.9418**	**0.0769**	**0.9458**	**0.0816**
**5-ary LRT**	**0.9700**	**0.0432**	**0.9141**	**0.1035**
**7-ary LRT**	**0.9761**	**0.0625**	**0.8936**	**0.1075**
matched filter	0.9567	0.0596	0.8915	0.1458

However, this analysis could be biased because it may depend on the number of pairs *S*, 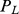 used to compute the averages and standard deviation, the non-uniform location of these pairs, etc.

### Receiver Operating Characteristic Curves

The receiving operating characteristic (ROC) curves have shown to be very effective for the evaluation of any kind of detector [3], [14], [15]. They actually test the robustness of the system by showing the tradeoff between the error probabilities of QRS segments and non-QRS detection as the threshold varies and completely describe the detector error rate. However the performance of the detector depends on the background SNR and therefore, the ROC curves are obtained by averaging the performance of the detector on every single record. Thus, the average should be computed under the same conditions and the ROC curves should be displayed on several conditions, i.e. SNR, type of noises etc. in order to provide a fair comparison.

**Figure 7:**
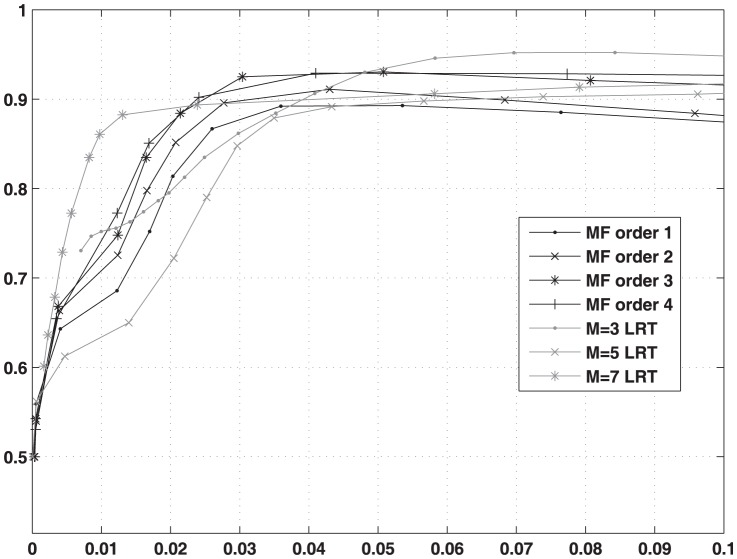
Zoom on the ROC curves by averaging all the tapes in the database. Results using the same observation interval are plotted for the two MAP strategies. The dependence of the operation point 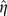 on the noise level affects the performance of all detectors.

Nevertheless, figure 7 shows the 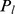 versus the false alarm rate (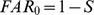) for all the records of the MIT-BIH database under several noise conditions. Additionally, to help the interpretation of the results, table 2 shows the Area Under Curve (AUC) for the ROC curves in figure 7. The proposed method yields better results than the previous MAP method for *M* = 7 and similar results for other detector configurations. The improvements of all MAP detectors are provided by the robustness of the decision rule of these statistical methods that are developed with a suitable design.

**Table 2:** Area Under Curve (AUC) of the ROC curves obtained by averaging all the tapes in the database.

	Complete curve	Zoomed region
**MF order 1**	0.7490	0.7355
**MF order 2**	0.7668	0.7270
**MF order 3**	0.7726	0.7916
**MF order 4**	0.7584	0.7836
**M = 3 LRT**	0.8299	0.8593
**M = 5 LRT**	**0.8524**	**0.8707**
**M = 7 LRT**	0.7916	0.8033

### Complete QRS performance study

For the above-mentioned reasons many papers evaluate the proposed detectors on standard or non-standard databases [3], by limiting their study to the inclusion of a tape-by-tape performance list. One example of a standard database is the MIT-BIH database [29] where several examples of ECG signals and noises are collected and become a real challenge. Another option is to collect own databases [14], [15], the so-called non standard databases, with controlled noise conditions which allow to fairly evaluate the performance of the proposed systems. In order to allow for comparisons, our approach is tested against a free-access standard database [20]. As in previous sections, the comparison is focused on the decision rule stage using the matched-filter based detector as a baseline framework.

Table 3 shows the same analysis as in [29]. The proposed detector produces 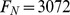 false negative detections and 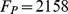 false positive detections for a sensitivity of 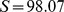 percent and a positive likelihood of 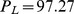 percent. This includes all episodes of ventricular flutter and other difficult segments that occur on several tapes, i.e. 207. The results for the matched filter are similar to the ones obtained by our method, i.e. over the 112646 beats the baseline detector produces 3910 false positives and 2749 false negatives for a 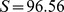 and a 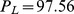. The positive false alarms that occurred were most often caused by the existence of tall and peaked T waves. This is due to their spectral properties that differ so little from those of the QRS complex. The negative false alarms were most often caused by the wrong adaptation of the noise model preceded by a frame where a QRS detection failure occurred. Again, it is worth mentioning that the detectors do not include any other linear filtering or nonlinear transformation stages that of course could improve the performance of both detectors.

**Table 3:** Results of real time evaluation with the M([1], [3], [5], [7], [9])-ary LRT Detector (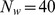, 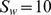).

Tape	Total (beats)	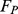	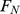	Failed detection ( 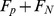 )	Failed detection ( 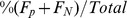 )
100	2274	0	2	2	0.087951
101	1874	4	6	10	0.53362
102	2192	0	5	5	0.2281
103	2091	0	8	8	0.38259
104	2311	49	121	170	7.3561
105	2691	132	120	252	9.3645
106	2098	32	94	126	6.0057
107	2140	9	30	39	1.8224
108	1824	154	50	204	11.1842
109	2535	0	3	3	0.11834
111	2133	17	5	22	1.0314
112	2550	1	13	14	0.54902
113	1796	6	2	8	0.44543
114	1890	12	18	30	1.5873
115	1962	2	7	9	0.45872
116	2421	8	29	37	1.5283
117	1539	6	3	9	0.5848
118	2301	0	25	25	1.0865
119	2094	7	108	115	5.4919
121	1876	7	11	18	0.95949
122	2479	1	1	2	0.080678
123	1519	0	1	1	0.065833
124	1634	1	15	16	0.97919
200	2792	138	204	342	12.2493
201	2039	74	66	140	6.8661
202	2146	5	21	26	1.2116
203	3108	321	252	573	18.4363
204	2672	22	18	40	1.497
205	2385	122	449	571	23.9413
207	3040	47	108	155	5.0987
208	3052	14	42	56	1.8349
209	2685	33	36	69	2.5698
210	2763	21	14	35	1.2667
212	3294	8	49	57	1.7304
213	2297	21	36	57	2.4815
214	3400	10	41	51	1.5
215	2280	28	106	134	5.8772
217	2312	5	160	165	7.1367
219	2068	0	20	20	0.96712
220	2462	24	31	55	2.234
221	2634	192	265	457	17.35
222	2643	91	43	134	5.07
223	2141	149	101	250	11.6768
228	2466	10	209	219	8.8808
230	2011	250	10	260	12.9289
231	1816	117	20	137	7.5441
232	3152	7	84	91	2.8871
233	2764	1	10	11	0.39797
**TOTAL**	**112646**	**2158**	**3072**	**5230**	**4.6429**

The QRS decision rule is formulated over a sliding window consisting of 2L+1 observation feature vectors around the frame 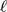 for which the decision is being made. This strategy, known as long term information provides very good results using several approaches for detection [30], however it imposes an m-frame delay on the algorithm that, for several applications including QRS detection, is not a serious implementation obstacle. As an example for the case 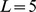, 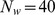 and 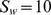 and 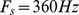, the delay of the algorithm is 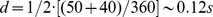.

## Conclusions

The use of a M-ary statistical LRT based approach to QRS detection was analyzed in this paper. The method uses a M-ary LRT defined over a multiple observation window in the Fourier domain and, additionally, it reduces the complexity of the signal model proposed in previous approaches. We proposed an alternative model based on the independent Gaussian properties of the DFT coefficients which simplifies the MAP probability function. One of its main strengths is that the observation interval is modeled as a discontinuous transmission discrete-time stochastic process which avoids the inclusion of parameters that usually constraint the morphology of the QRS complexes. Furthermore, other methods require the estimation of parameters that are essentially time dependent. On the contrary, in the proposed method, the only parameters that need to be estimated are the variances of the noise and the desired signal. Another important fact is that, unlike other approaches, we use the same detector structure with the same parameter values in all the phases of the algorithm.

After the description and derivation of the proposed algorithm, we presented a simplification in order to demonstrate the ability of the M-LRT to effectively discriminate between QRS and noise frames for a wide range of SNRs during step transitions in the observation window. Moreover, we preliminarily discussed the derivation of a potential improvement to the proposed algorithm which includes the window size of the observation in the maximization so it is adjusted to the most probable hypotheses. Future work will be oriented to study the performance of such configuration.

Regarding the performance of the proposed algorithm, we fairly compared it to a real implementation of the classical matched filter method. To allow for future comparison of our method, we tested our method over the standard and publicly available MIT-BIH database, which includes different QRS morphologies, types of noise and SNRs.

It is also important to remark that since the goal of the paper was to propose a new algorithm for the decision stage, the analyzed detectors did not include any other linear filtering or nonlinear transformation stage that could have improved their overall performance. Moreover the classical method used as a baseline required the selection of a perfect time-reversed desired waveform to effectively perform in QRS detection, among a large number of parameters [14]. On the other hand, by defining a suitable observation interval, the proposed detector provided similar detection rates but under easier parameter tuning conditions.

We showed that, while the revised method yields similar QRS detection accuracy when compared to the matched filter based detector at a low model order, it exhibits an improved accuracy in detecting QRS periods when the order is increased to M = 7. The improvements of the proposed method are especially important for poor SNRs and the presence of artifacts or abnormal QRS shapes. Furthermore, it was shown that the M-ary LRT method yields a significant improvement in both the sensitivity (*S*) and the positive likelihood (*P_L_*) when the model order is greater than M = 3. Among the different tested configurations, the proposed detector scheme for M = 5 achieves the best compromise between *S* and *P_L_*.

## Supporting Information

Appendix S1 (TEX)
