# Correction: Real Time QRS Detection Based on M-ary Likelihood Ratio Test on the DFT Coefficients

**DOI:** 10.1371/journal.pone.0116654

**Published:** 2014-12-26

**Authors:** The PLOS ONE Staff

Appendix S1 appears incorrectly. Please view the complete, corrected Appendix S1 below.

## Supporting Information

Appendix S1 (PDF)
