# Supplementary material for: Correction: Real Time QRS Detection Based on M-ary Likelihood Ratio Test on the DFT Coefficients
Source: PLoS One. 2014 Dec 26;9(12):e116654. doi: 10.1371/journal.pone.0116654 (PMC4277461; doi:10.1371/journal.pone.0116654)
Supplement: Appendix S1 — (PDF) [file pone.0116654.s001.pdf]

Appendix: Calculation of $p(\mathbf{h})$

In order to evaluate the LRT in Equation (13) we need to compute the *a priori* probabilities of the states $\mathbf{P}_k$. Among all the 2^{2L+1} possible hypotheses on the observation window of size $2L+1$ only a few of them could be observed due to the shape of the incoming ECG signal. In particular we assume that the length of the observation window is reduced enough so two QRS complexes do not occur within the buffer (small observation window approximation).

Case $Q \geq L$: The probability of the $\mathbf{h}$ hypotheses with any transition is easily obtained as the number K of QRS blocks in the data sample divided by the total number of frames F, that is, the probability of QRS segments $\phi = K/F$. For the hypothesis $\mathbf{h} = \mathbf{1}$, the number of cases is equal to the number of QRS frames S minus the number of all the situations corresponding to transitional hypotheses assuming a 1 hypothesis in the analysis window ($2(L-r)$ per QRS block, where r is the radius of the closed ball $\mathfrak{B}_r(L+1)$), that is,

$$p(\mathbf{h}) = [S - 2(L-r)K]/F = \rho - 2(L-r)\phi \tag{1}$$

In a similar way, for $\mathbf{h} = \mathbf{0}$, $p(\mathbf{h}) = [(F-S) - 2(L+r)K]/F = 1 - \rho - 2(L+r)\phi$. Thus, the set of probabilities yields:

$$p(\mathbf{h}) = \begin{cases} \phi & ;\ \mathbf{h} \in \mathbf{H} \wedge \mathbf{h} \neq \mathbf{0} \wedge\ \mathbf{h} \neq \mathbf{1} \\ \rho - 2(L-r)\phi & ;\ \mathbf{h} = \mathbf{1} \\ (1-\rho) - 2(L+r)\phi & ;\ \mathbf{h} = \mathbf{0} \\ 0 & ;\ \mathbf{h} \notin \mathbf{H} \end{cases}$$

where $\rho \equiv S/F$ is the *a priori* probability of the QRS frames. The set of hypotheses can be described as a $2(2L+1) \times (2L+1)$ Hankel matrix, so that each row identifies a global hypothesis as shown in the following.

Examples

Let assume $L = 1 < Q = 2$, $\mathfrak{B}_0[L+1]$ and the buffer $\widehat{\mathbf{X}}$ with associated hypotheses $\hat{\mathbf{h}}$:

$$\hat{\mathbf{h}} = \{...00011111000...\} \tag{2}$$

The selected observation window shows $F = 11$, $S = 5$ frames and $K = 1$ QRS segments. The set of hypotheses are given by:

$$\mathbf{H} = \begin{bmatrix} \mathbf{H}_1 \\ \mathbf{H}_0 \end{bmatrix}; \mathbf{H}_1 = \begin{bmatrix} 0 & 1 & 1 \\ 1 & 1 & 1 \\ 1 & 1 & 0 \end{bmatrix}; \mathbf{H}_0 = \begin{bmatrix} 0 & 0 & 0 \\ 0 & 0 & 1 \\ 1 & 0 & 0 \end{bmatrix}$$

Case $Q < L$: Short time duration waveforms such as the QRS complexes require a model that handles the possibility of more than one transition. Thus, if $L > Q$ the hypothesis $\mathbf{h} = \mathbf{1}$ is not present in the observation window and its probability is null. The rest of probabilities are defined by the following relation:

$$p(\mathbf{h}) = \begin{cases} \phi & ;\ \mathbf{h} \in \mathbf{H} \wedge \mathbf{h} \neq \mathbf{0} \\ (1-\rho) - 2(L+r)\phi & ;\ \mathbf{h} = \mathbf{0} \\ 0 & ;\ \mathbf{h} \notin \mathbf{H} \end{cases} \tag{3}$$

In addition, as the selection criteria considers $\mathfrak{B}_r[L+1]$, the $[2(Q+L+1)] \times (2L+1)$ Hankel matrix of hypotheses is[1]:

$$\mathbf{H} = \begin{bmatrix} 0 & 0 & \cdots & 0 & \cdots & 0 & 0 \\ 0 & 0 & \cdots & 0 & \cdots & 0 & 1 \\ 0 & 0 & \cdots & 0 & \cdots & 1 & 1 \\ \vdots & \vdots & \vdots & \vdots & \vdots & \vdots & \vdots \\ 0 & 0 & \cdots & 1 & \cdots & 1 & 1 \\ & & \leftarrow & 2Q+1 & & \rightarrow & \end{bmatrix} \begin{bmatrix} 0 & \cdots & 1 & 1 & \cdots & 1 & 0 \\ \vdots & \vdots & \vdots & \vdots & \vdots & \vdots & \vdots \\ 1 & 1 & \cdots & 1 & \cdots & 0 & 0 \\ \vdots & \vdots & \vdots & \vdots & \vdots & \vdots & \vdots \\ 1 & 1 & \cdots & 0 & \cdots & 0 & 0 \\ 1 & 0 & \cdots & 0 & \cdots & 0 & 0 \end{bmatrix} \tag{4}$$

As shown in (4) the number of states in $\mathbf{H}_1$ is $2(Q-r)+1$ and in $\mathbf{H}_0$ is $2(L+r)+1$.

[1] Represented in two columns for clarity reasons

Now, let assume $L = 2 > Q = 1$ and $\mathfrak{B}_1[L+1]$, thus:

$$\mathbf{H} = \begin{bmatrix} \mathbf{H}_1 \\ \mathbf{H}_0 \end{bmatrix}; \mathbf{H}_1 = \begin{bmatrix} 0 & 1 & 1 & 1 & 0 \end{bmatrix}; \mathbf{H}_0 = \begin{bmatrix} 0 & 0 & 0 & 0 & 0 \\ 0 & 0 & 0 & 0 & 1 \\ 0 & 0 & 0 & 1 & 1 \\ 0 & 0 & 1 & 1 & 1 \\ 1 & 1 & 1 & 0 & 0 \\ 1 & 1 & 0 & 0 & 0 \\ 1 & 0 & 0 & 0 & 0 \end{bmatrix}$$
